# Supplementary material for: Bioadhesive and conformable bioelectronic interfaces for vasomotoricity monitoring and regulation
Source: Nat Commun. 2025 Oct 14;16:9103. doi: 10.1038/s41467-025-64118-2 (PMC12521587; doi:10.1038/s41467-025-64118-2)
Supplement: Supplementary file 1 — Supplementary Information [file 41467_2025_64118_MOESM1_ESM.pdf]

Supplementary Materials for

Bioadhesive and Conformable Bioelectronic Interfaces for Vasomotoricity Monitoring and Regulation

Xiner Wang[1,2†], Weijian Fan[3†], Yuxin Liu[1,2], Li Chen[4], Erda Zhou[1,2], Xiaoling Wei[2,5,6], Liuyang Sun[1,2,6], Bo Yu[3], Tiger H. Tao[7,8,9*], Zhitao Zhou[2,5,6*], Jinyun Tan[3*]

[1]2020 X-Lab, Shanghai Institute of Microsystem and Information Technology, Chinese Academy of Sciences, Shanghai 200050, China.

[2]School of Graduate Study, University of Chinese Academy of Sciences, Beijing 100049, China.

[3]Department of Vascular Surgery, Huashan Hospital of Fudan University, Shanghai 200040, China.

[4]Department of Ultrasound, Huashan Hospital of Fudan University, Shanghai 200040, China.

[5]State Key Laboratory of Transducer Technology, Shanghai Institute of Microsystem and Information Technology, Chinese Academy of Sciences, Shanghai 200050, China.

[6]School of Integrated Circuits, University of Chinese Academy of Sciences, Beijing 100049, China.

[7]Neuroxess Co., Ltd., Shanghai 200023, China.

[8]Guangdong Institute of Intelligence Science and Technology, Hengqin, Zhuhai, Guangdong 519031, China.

[9]Tianqiao and Chrissy Chen Institute for Translational Research, Shanghai, China.

*Corresponding author. Email: tiger@mail.sim.ac.cn; ztzhou@mail.sim.ac.cn; m.tan@fudan.edu.cn

†These authors contributed equally to this work.

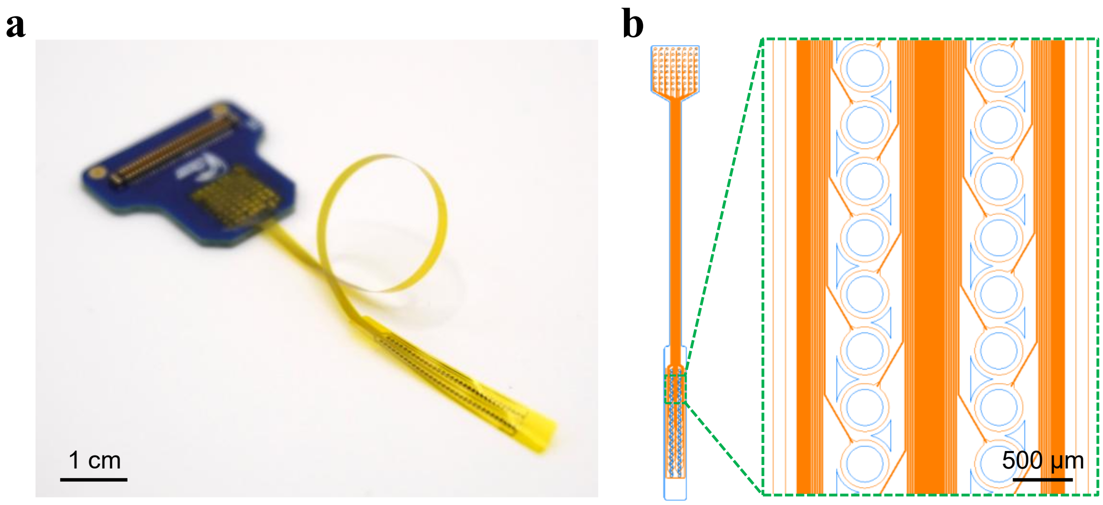

Supplementary Figure 1. Design of the BACE interface. a, Photograph of the device, emphasizing its flexibility. The interface is bonded to a printed circuit board (PCB), with connectors facilitating externalization. **b**, Layout of the multi-channel interface. Each device comprises 64 recording sites, each with a diameter of 300 μm.

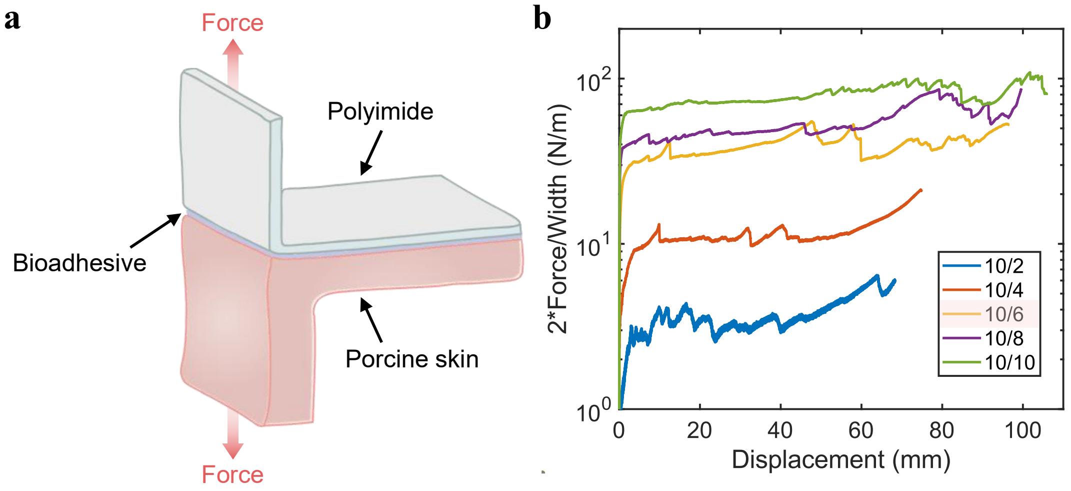

Supplementary Figure 2. Adhesion performance of the bioadhesive. a, Schematic illustration of the 180° peel test (ASTM F2256) for interfacial toughness measurement between the porcine skin and polyimide. **b**, 2*Force/width vs. displacement curves for the 180° peel test adhered by the SF/PU composites with various ratios. The selected ratio is shaded in pink.

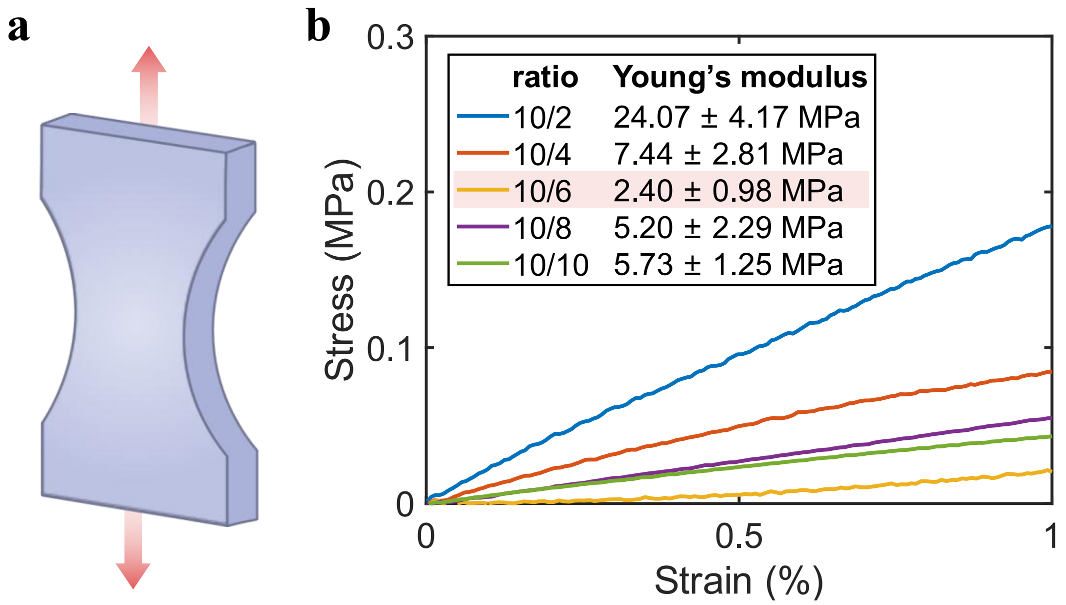

Supplementary Figure 3. Tensile performance of the SF/PU composites. a, Schematic representation of the tensile test. **b**, Close-up view of representative stress-strain curves for SF/PU composites with varying ratios, illustrating differences in Young's modulus. All the samples were prepared and equilibrated in 1x PBS at room temperature for 24 hours before testing. The selected ratio is shaded in pink.

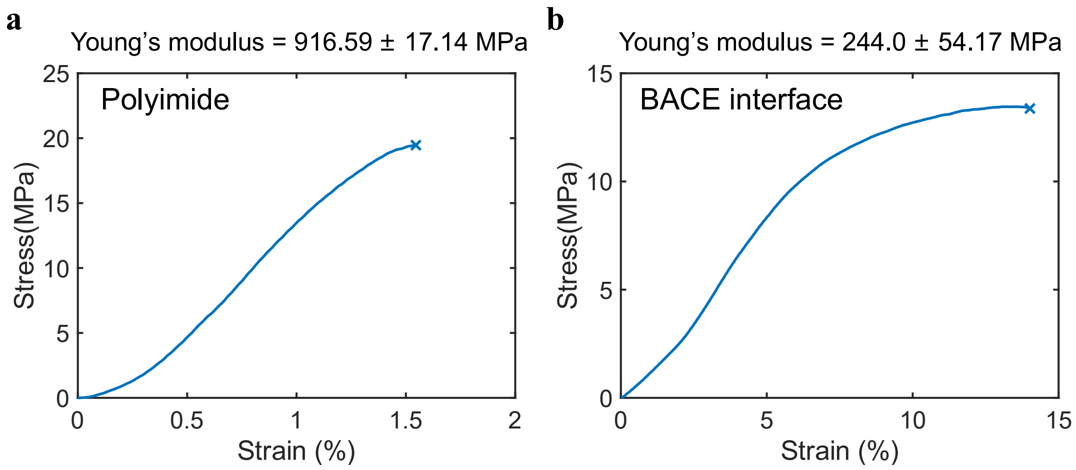

Supplementary Figure 4. Tensile performance of the bare polyimide and BACE interface. a, Representative stress-strain curve for the bare polyimide, with the cross indicating the breaking point. **b**, Representative stress-strain curve for the BACE interface, with the cross indicating the breaking point. Reproducibility was ensured through measurements from n = 3 samples. Young's modulus is presented as mean ± SD.

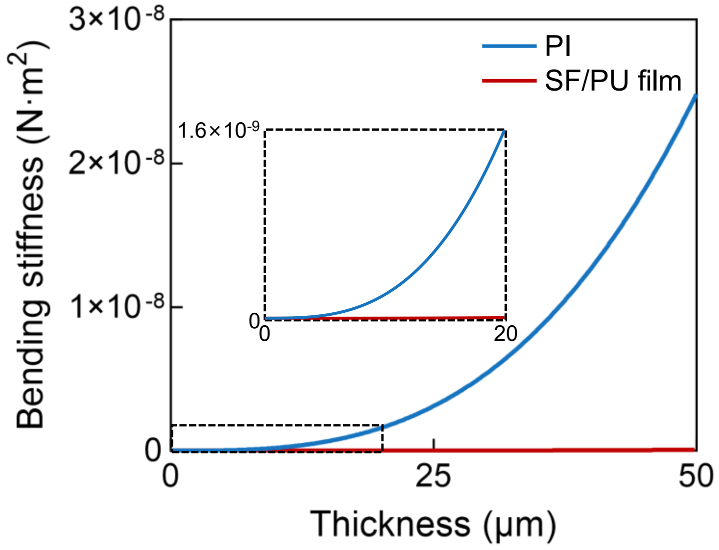

Supplementary Figure 5. Comparative analysis of the bending stiffness. The bending stiffness of bare polyimide (blue) and the SF/PU film (red) as a function of thickness. Inset: Enlarged view of the curves of these two substrates for the thickness range of 0-20 μm. Notably, the bending stiffness of the interface based on bare polyimide was calculated as follows[56,57]:

$$\mathrm{EI} = E_{\mathrm{PI}} w_p t_p \left(\frac{1}{3} t_p^2 - t_p h + h^2\right) + (E_m - E_{\mathrm{PI}}) w_m t_m \left[\frac{1}{3} t_m^2 + t_m(t' - h) + (t' - h)^2\right] \tag{1}$$

while the bending stiffness of the interface with a supporting silk substrate is:

$$\mathrm{EI} = E_{\mathrm{PI}} w_p t_p \left(\frac{1}{3} t_p^2 - t_p h + h^2\right) + E_{\mathrm{silk}} w_s t_s \left(\frac{1}{3} t_s^2 + t_s h + h^2\right)$$
$$+ (E_m - E_{\mathrm{PI}}) w_m t_m \left[\frac{1}{3} t_m^2 + t_m(t' - h) + (t' - h)^2\right] \tag{2}$$

E_{PI}, E_m, and E_{silk} represent Young's modulus of polyimide, metal, and silk substrate, respectively. w_p, w_m, and w_s denote the total widths of polyimide, metal, and silk substrate, while t_p, t_m, and t_s indicate their respective thicknesses. h is the distance between the neutral axis and the bottom of the interface, and t' is the distance between the bottom of the metal layer and the polyimide. Calculations reveal that the bending stiffness of a PI interface with a thickness of 20 μm is 1.6811×10^{-9} N m^2, while the total bending stiffness of a 40 μm interface supported by silk films is 1.6854×10^{-9} N·m^2. This indicates that the SF/PU composite, with its low Young's modulus, enhances the interface adhesion and mechanical compliance without compromising bending stiffness.

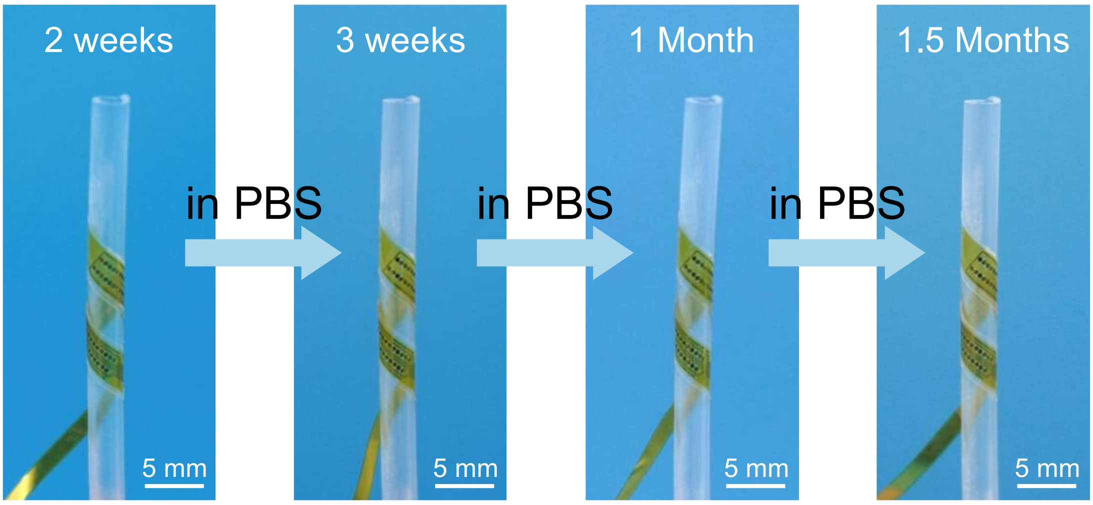

Supplementary Figure 6. Stable and robust adhesion in aqueous environment. A series of photographs demonstrate that the BACE interface conformally wrapped around a cylinder tube immersed in 1x PBS. These images were captured midway through the two-month immersion period described in Fig. 1d, showcasing the interface's durability and strong adhesion under prolonged aqueous conditions.

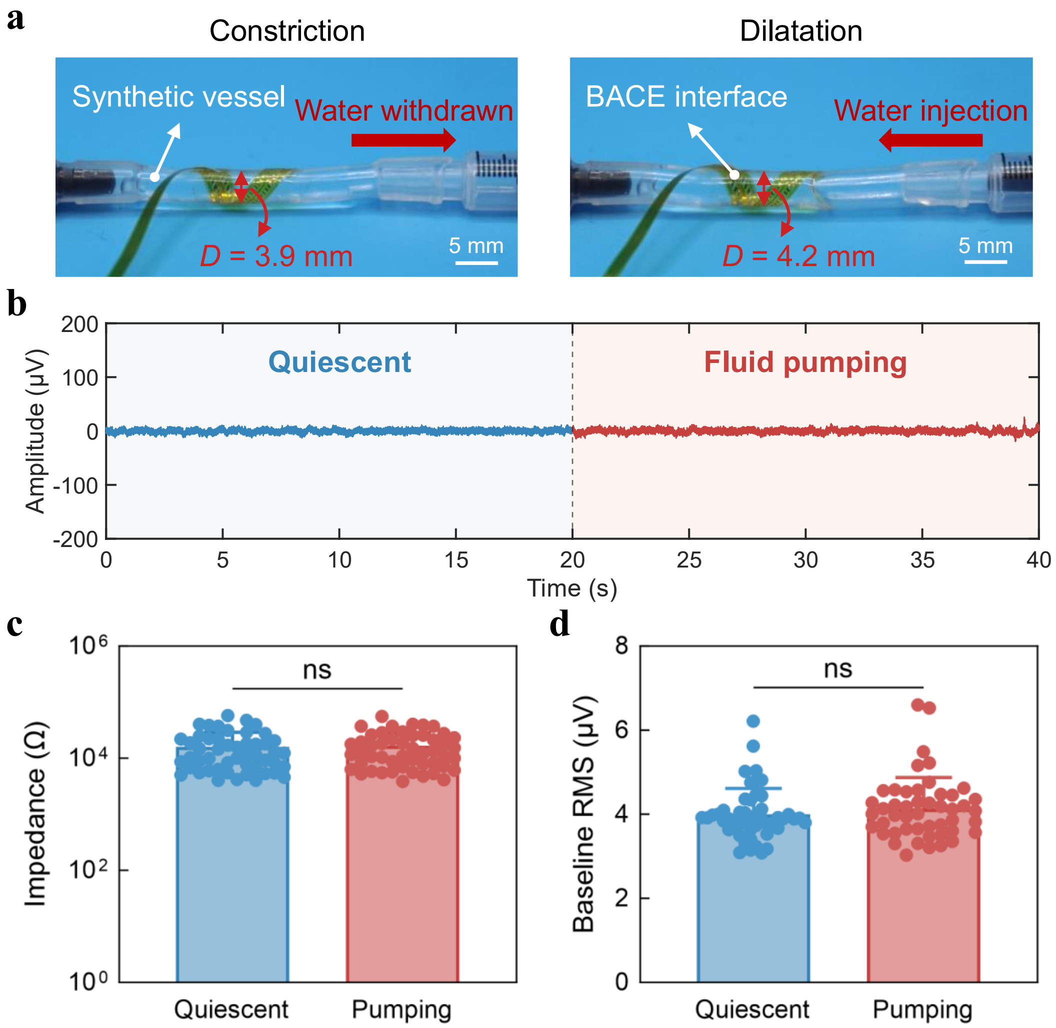

Supplementary Figure 7. *In vitro* experiment simulating vasoconstriction and vasodilation. a, Experimental setup showing the BACE interface wrapped around a synthetic vessel. Water is withdrawn and injected to simulate vessel contraction and dilation. Red arrows indicate the direction of fluid movement. **b**, Representative electrophysiological traces recorded during a quiescent period and during cyclic fluid pumping. The baseline signal remained stable with minimal amplitude drift when the interface is subjected to fluid-induced pressure. **c**, Impedance measurements during the phase of quiescence and fluid pumping. **d**, Quantitative comparison of baseline signal RMS amplitudes under quiescent and pumping conditions. Data in **c** and **d** are presented as mean values ± SD. p values for data in **c** and **d**: $p = 0.9234$ (impedance comparison) and $p = 0.4656$ (baseline RMS comparison). (ns, : $p > 0.05$, $*p < 0.05$, $**p < 0.01$, $***p < 0.001$, $****p < 0.0001$, Wilcoxon signed-rank test)

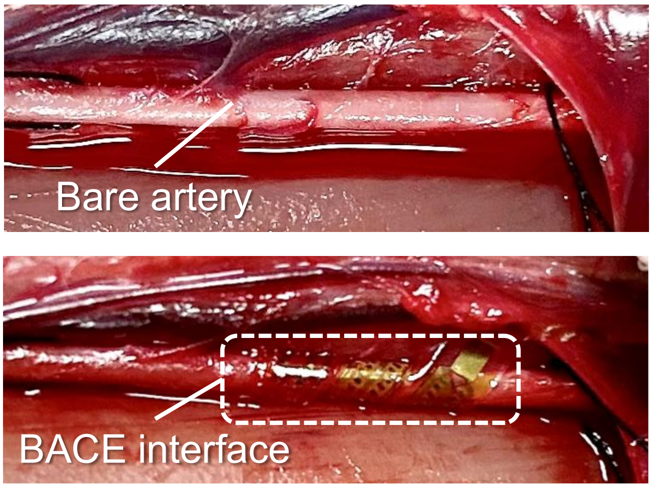

Supplementary Figure 8. Conformal wrapping around the artery. Optical images showing the exposed bare abdominal aorta of a rabbit (top) and the same aorta wrapped with the BACE interface (bottom). The BACE interface conforms closely to the arterial surface without exerting any constriction on the vessel or disrupting blood flow.

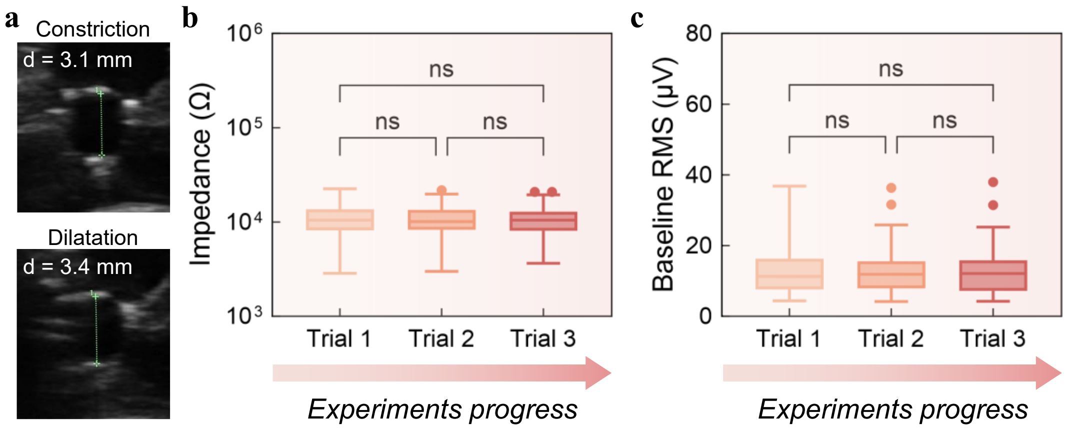

Supplementary Figure 9. Reliability and stability of the wrapping around the artery. a, The ultrasound image of the abdominal aorta wrapped with the BACE interface indicates its normal expansion by the measured systolic and diastolic diameters. **b**, The interfacial impedance measured at 1 kHz during the three trials throughout the course of the experiment across $n = 3$ rabbits. **c**, The RMS amplitude of the baseline signals acquired from three trials on one rabbit as the experiment progresses. Data in **b** and **c** are presented as box plots which depict the data median (center line), upper and lower quartiles (box bounds), 1.5 times the interquartile range (whiskers) and outlier values beyond this range (circles). p values for impedance comparison: $p = 0.4508$ (Trial 1 vs. Trials 2), $p = 0.2154$ (Trial 1 vs. Trial 3), $p = 0.1258$ (Trial 2 vs. Trial 3). p values for amplitude comparison: $p = 0.1526$ (Trial 1 vs. Trials 2), $p = 0.4907$ (Trial 1 vs. Trial 3), $p = 0.0512$ (Trial 2 vs. Trial 3). (ns, $p > 0.05$, * $p \leq 0.05$, ** $p \leq 0.01$, *** $p \leq 0.001$, and **** $p \leq 0.0001$, Wilcoxon signed-rank test)

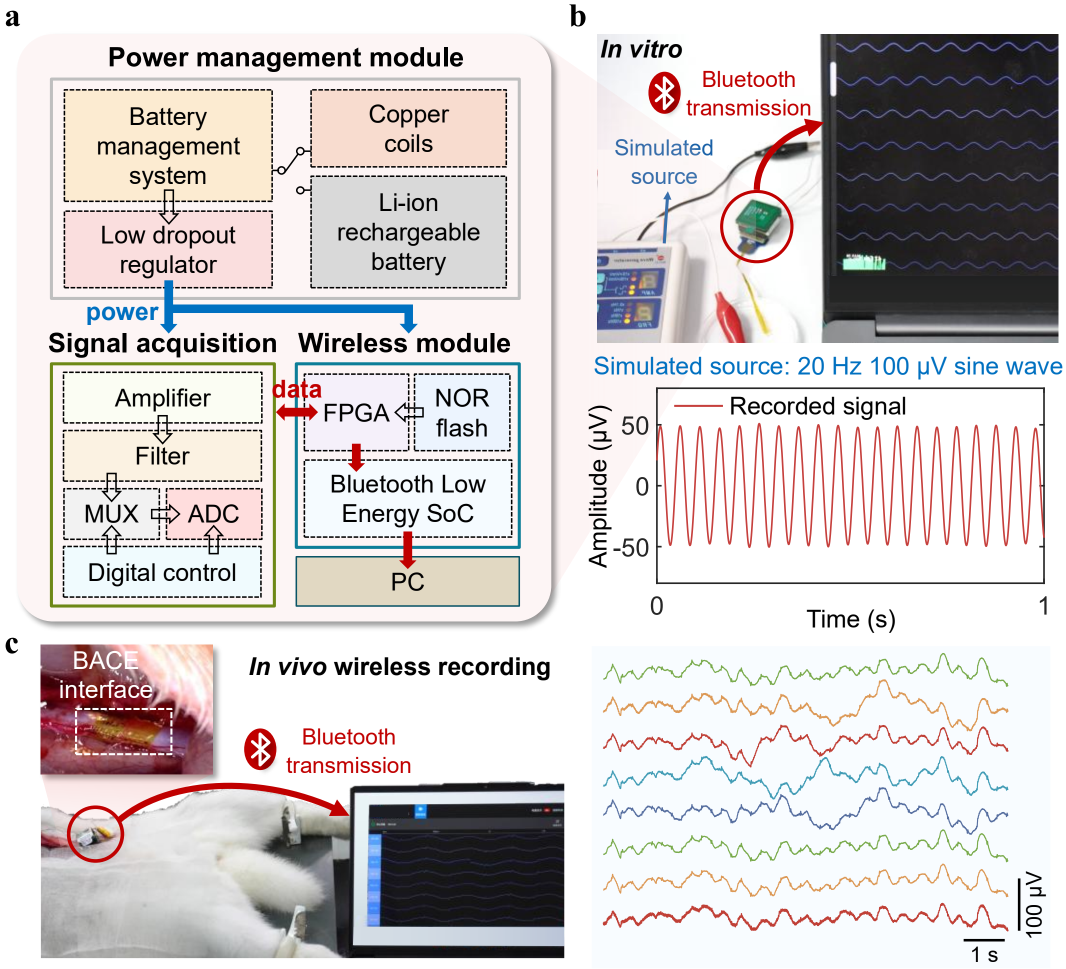

Supplementary Figure 10. Demonstration of the extended wireless data transmission system based on our BACE interface. a, Schematic diagram of the wireless system architecture, comprising the power management module, signal acquisition, and wireless module. **b**, *In vitro* functional validation of the wireless transmission system where a simulated 20 Hz, 100 μV sine wave is transmitted via Bluetooth, showing the output on the PC screen. **c**, *In vivo* wireless recording of the vascular electrophysiological signals acquired from the aorta of a rabbit via the BACE interface.

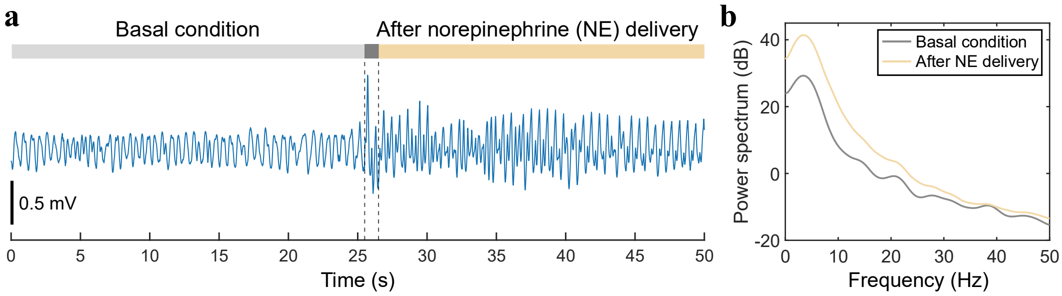

Supplementary Figure 11. Effects of exogenous norepinephrine delivery. a, Representative time-domain trace illustrating vascular electrical responses before and after exogenous NE delivery in an anesthetized rabbit, similar to Fig. 2b. **b**, Power spectral densities (PSDs) of VE signals recorded under basal conditions and following NE administration, highlighting changes in frequency-domain characteristics induced by NE delivery.

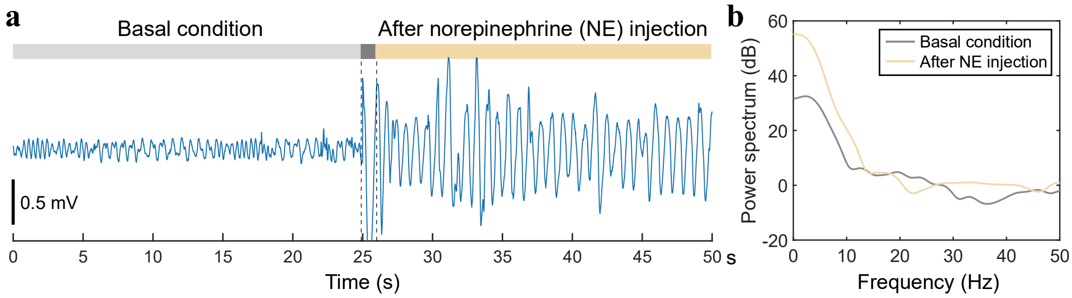

Supplementary Figure 12. Effects of intravenous norepinephrine injection. a, Representative time-domain trace demonstrating vascular electrical responses before and after intravenous NE injection in an anesthetized rabbit. **b**, PSDs of VE signals recorded under basal conditions and after NE injection, revealing a more pronounced response than exogenous NE delivery.

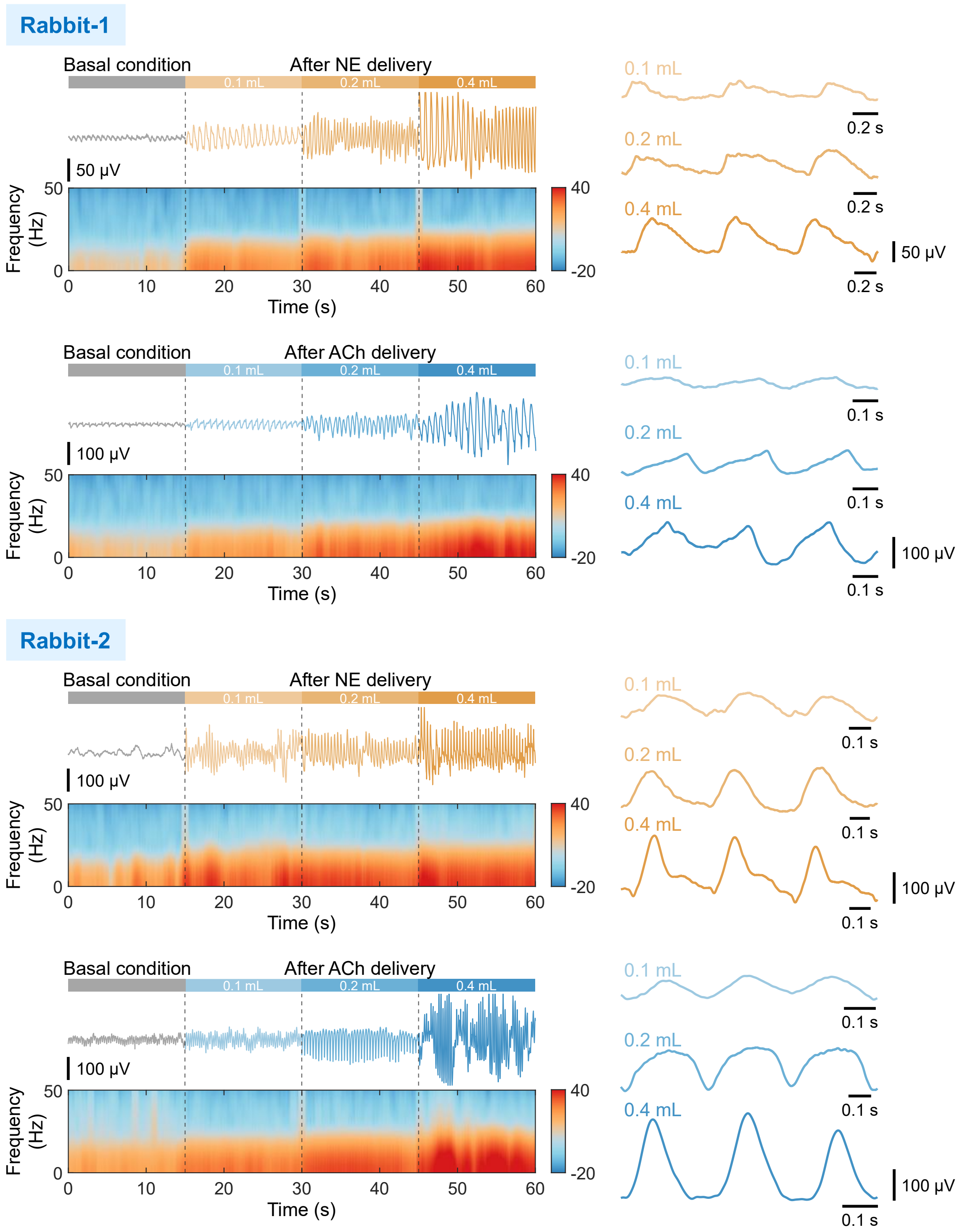

Supplementary Figure 13. Vascular electrical responses evoked by varying doses of different vasoactive agents. The representative time-domain waveforms and time-frequency spectrograms of the VE signals recorded under basal condition and after NE and ACh delivery from two rabbits (left) highlight the dose-dependent effects on vasomotor function. The zoomed-in view of the time-domain waveforms (right) provides a more detailed comparison of the amplitude and rhythm of the responses induced by different drugs at different doses.

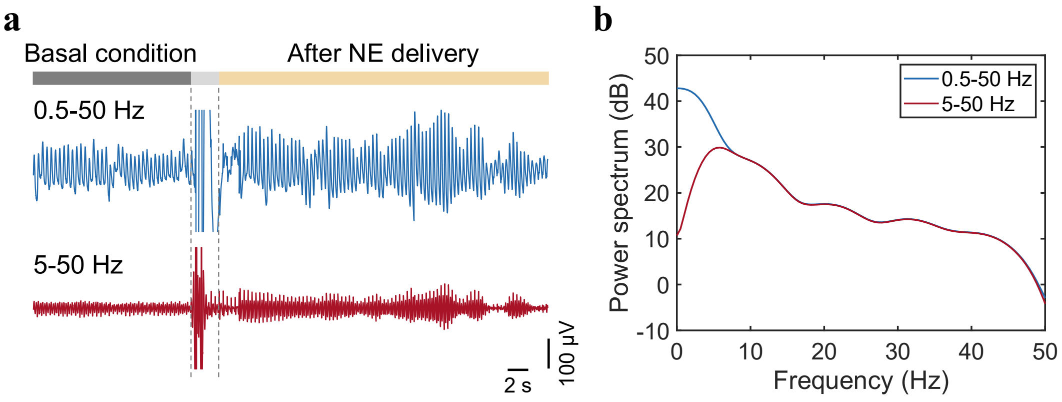

Supplementary Figure 14. Comparison of the original and filtered signals. a, Representative time-domain traces of the original signal (0.5-50 Hz) and the filtered signal (5-50 Hz) before and after NE delivery. **b**, Power spectral density (PSD) curves of the signals shown in **a** to verify the presence or absence of low-frequency components below 5 Hz.

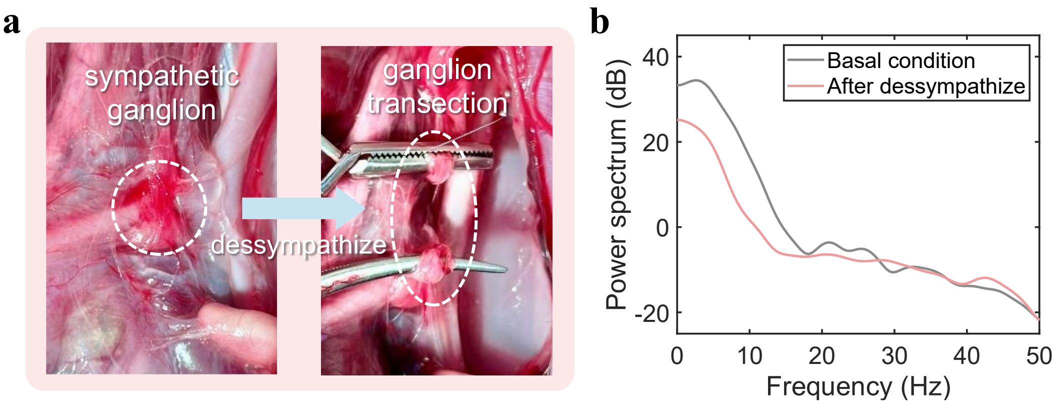

Supplementary Figure 15. Effects of sympathetic ganglion transection. a, Optical images illustrating the intact morphology of the sympathetic ganglion (left) and the transected ganglion (right). As the relay point between preganglionic and postganglionic sympathetic neurons, the sympathetic ganglion promotes the transmission of autonomic nerve signals. Consequently, ganglion transection significantly impairs the regulation of vasomotor function. **b**, PSDs of VE signals recorded under basal conditions and after dessympathize, showing an apparent suppression in vasomotor activity.

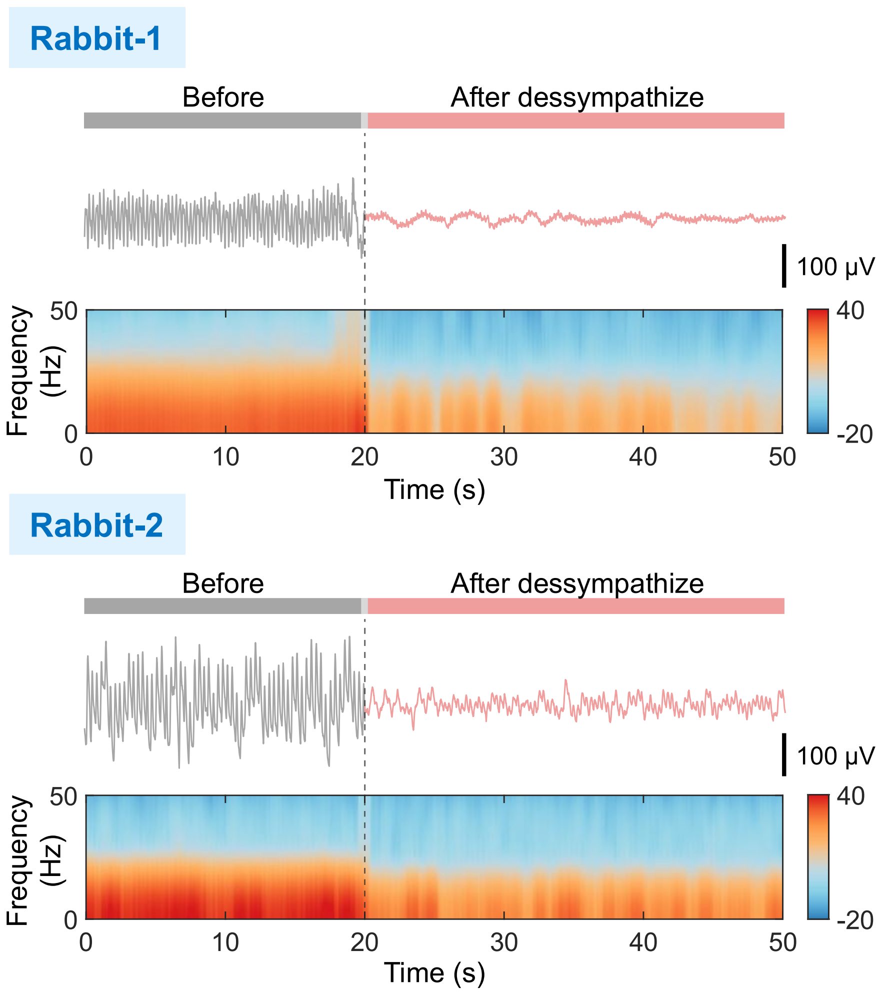

Supplementary Figure 16. Vascular electrical responses before and after sympathetic ganglionectomy. The representative time-domain waveforms and corresponding time-frequency spectrograms before and after severing the sympathetic path ways from two rabbits, indicative of the vasomotor function alteration.

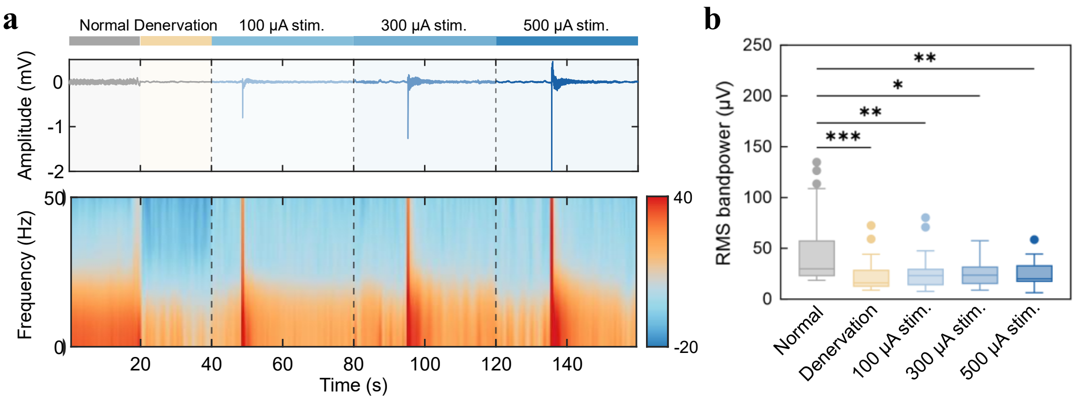

Supplementary Figure 17. Effects of electrical stimulation with different current intensities to the aorta after denervation. a, Representative time-domain waveforms and time-frequency spectrogram of the damaged vascular electrophysiological activity before and after electrical stimulation with intensities of 100, 300, and 500 μA. **b**, Quantitative analysis of vascular electrophysiological signal power under different conditions. Box plots in **b** depict the data median (center line), upper and lower quartiles (box bounds), 1.5 times the interquartile range (whiskers) and outlier values beyond this range (circles). p values for power comparison: $p = 2.848\times10^{-4}$ (Normal vs. Denervation), $p = 0.0053$ (Normal vs. 100 μA stimulation), $p = 0.0449$ (Normal vs. 300 μA stimulation), $p = 0.0028$ (Normal vs. 500 μA stimulation). (*$p < 0.05$, **$p < 0.01$, ***$p < 0.001$, ****$p < 0.0001$, Friedman test with Dunn's post hoc test).

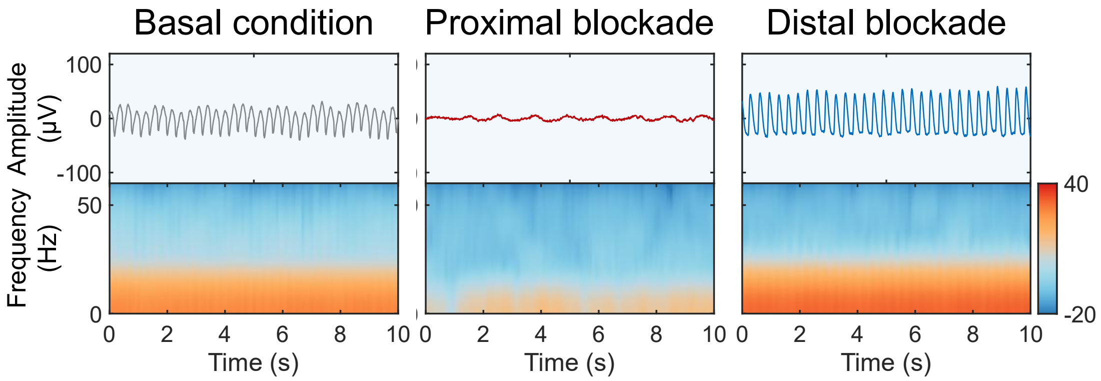

Supplementary Figure 18. Vascular electrophysiology monitoring in stenosis model. Representative channel illustrating vascular electrical activities in an anesthetized rabbit under three conditions: basal state, after the proximal blockade, and after the distal blockade. The data are presented as time-domain traces (top) and frequency-domain spectra (bottom).

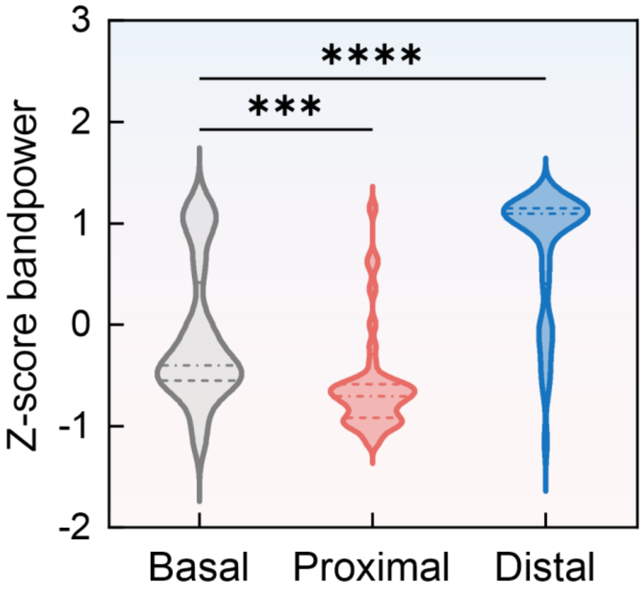

Supplementary Figure 19. Comparative analysis of the VE signals in stenosis model. The power within the 0-50 Hz frequency range of the vascular electrical signals from the three states shown in Supplementary Fig. 11 was extracted and normalized. Violin plots show data distribution with median (dot dashed line), and quartiles (dashed line) indicated. p values for power comparison: $p = 4.90\times10^{-4}$ (Basal condition vs. Proximal blockade), $p = 3.50\times10^{-5}$ (Basal condition vs. Distal blockade). (*$p < 0.05$, **$p < 0.01$, ***$p < 0.001$, ****$p < 0.0001$, Kruskal-Wallis test with Dunn's post hoc test).

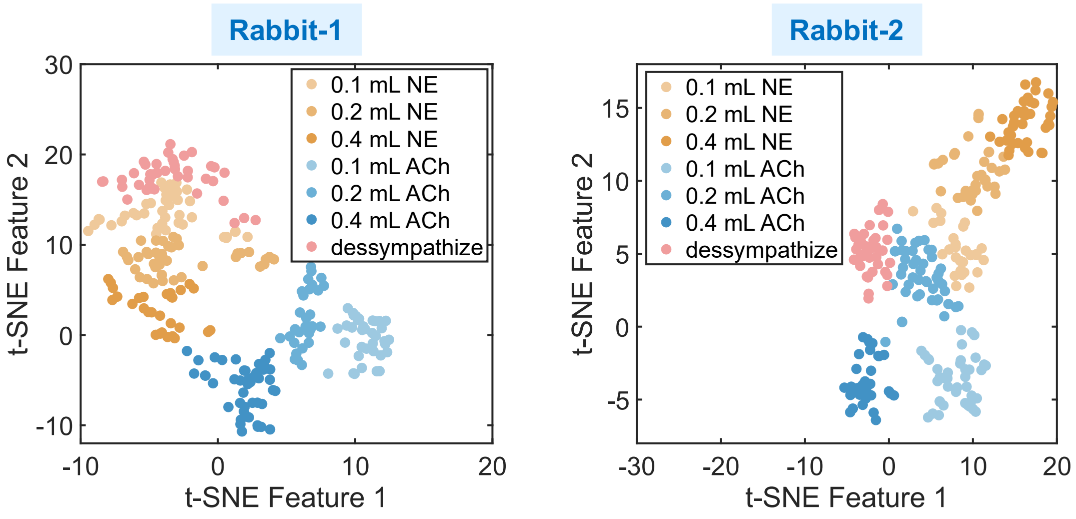

Supplementary Figure 20. Two-dimensional t-SNE feature distribution of the VE signals under pharmacological and surgical interventions. The plots illustrate the clustering of seven distinct electrophysiological features induced by varying doses of NE and ACh, as well as the effects of sympathetic ganglion transection, showing the separation between different vasomotor states of constriction and dilation.

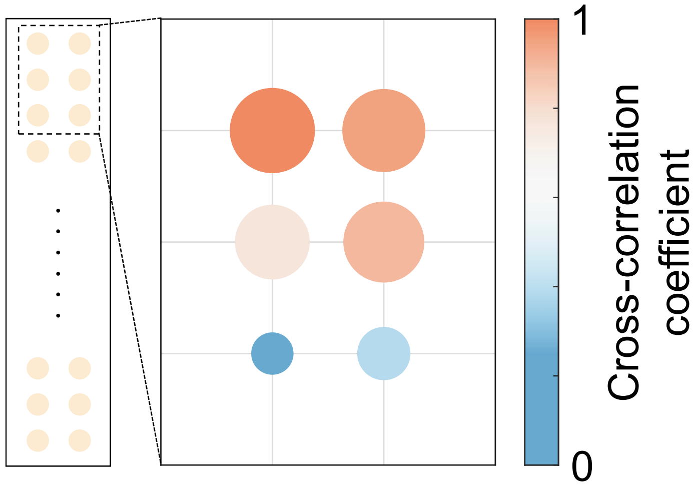

Supplementary Figure 21. Analysis of signal cross-correlation coefficient between adjacent channels within the density configuration of 64 channels.

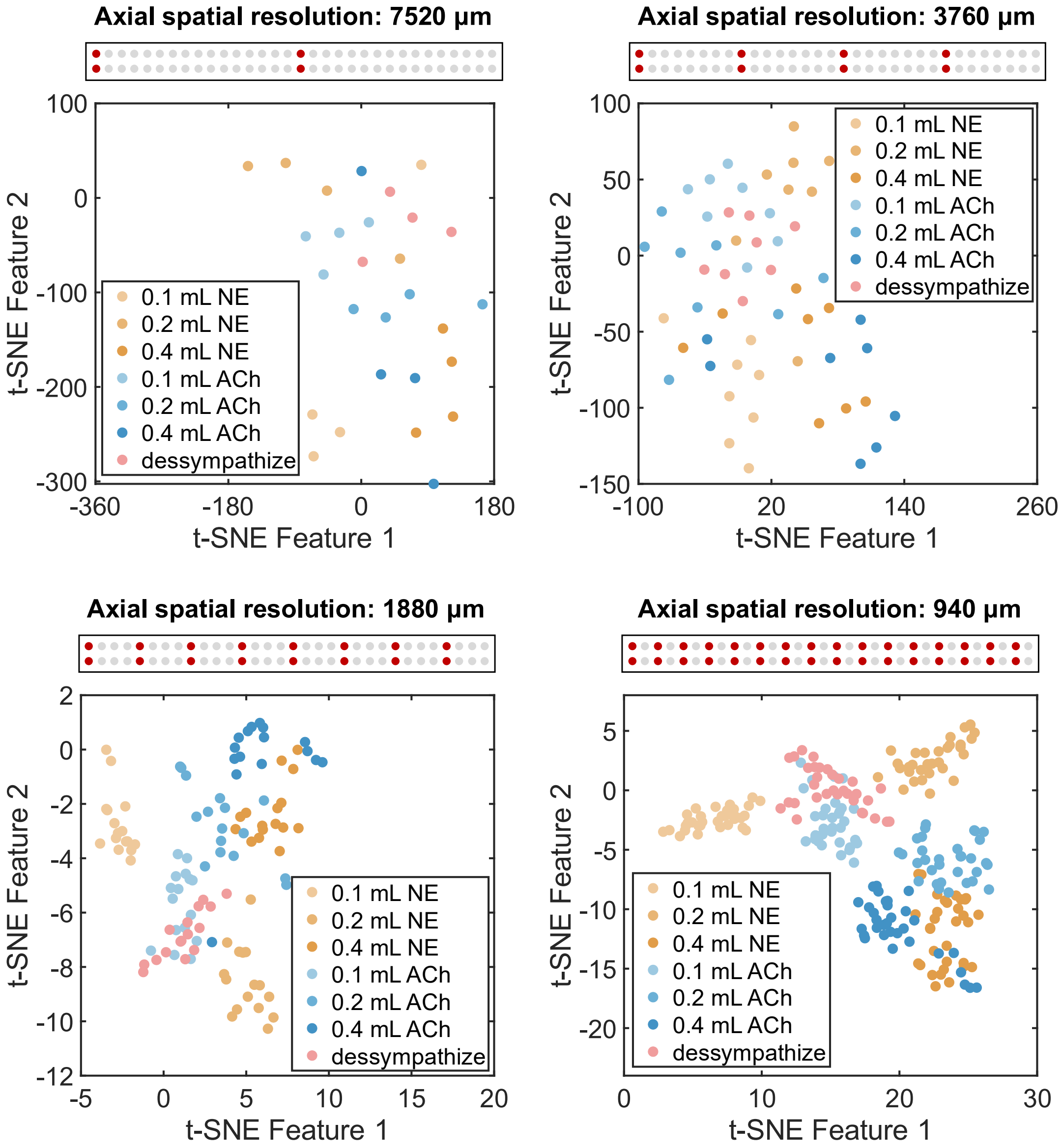

Supplementary Figure 22. *t*-SNE visualization of feature distributions extracted from datasets using different axial spatial resolutions. The subsets of 4, 8, 16, and 32 channels are selected varying the axial spatial resolution by adjusting the inter-electrode distance along the longitudinal direction within a fixed electrode array contour.

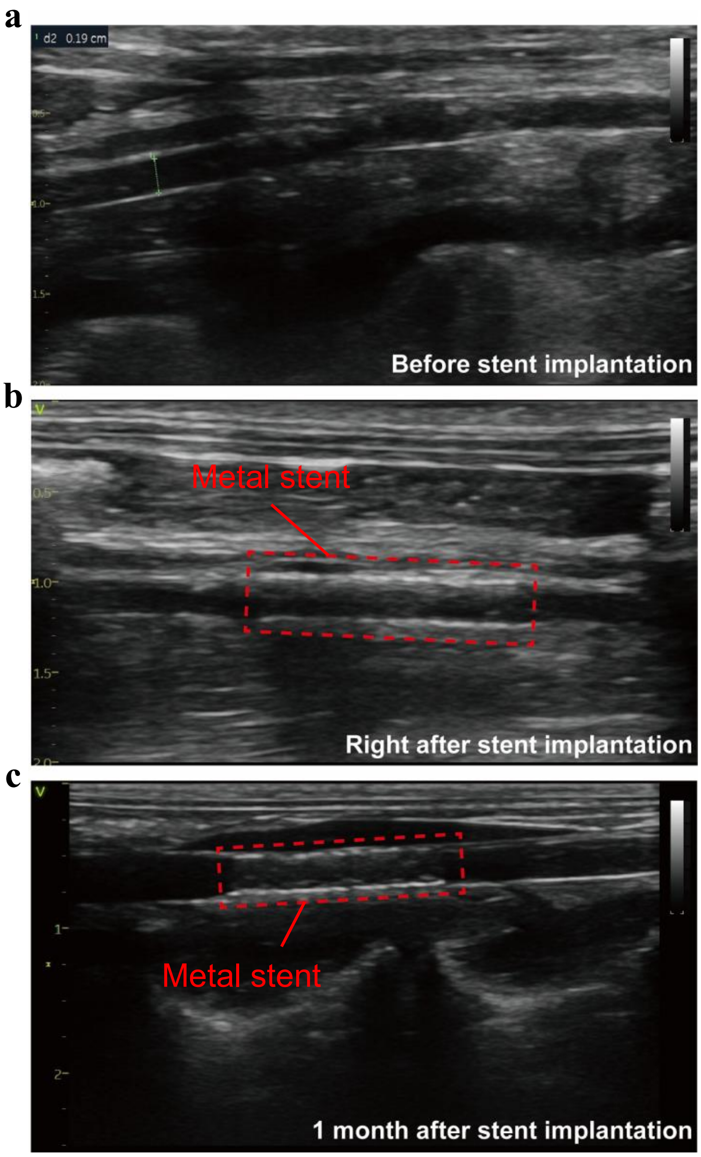

Supplementary Figure 23. Ultrasound visualization of the abdominal aorta in the stent model. Ultrasound images of the abdominal aorta in a rabbit prior to stent implantation (**a**), immediately after stent implantation (**b**), and one-month post-stent implantation (**c**). Red dashed boxes highlight the position of the metal stent.

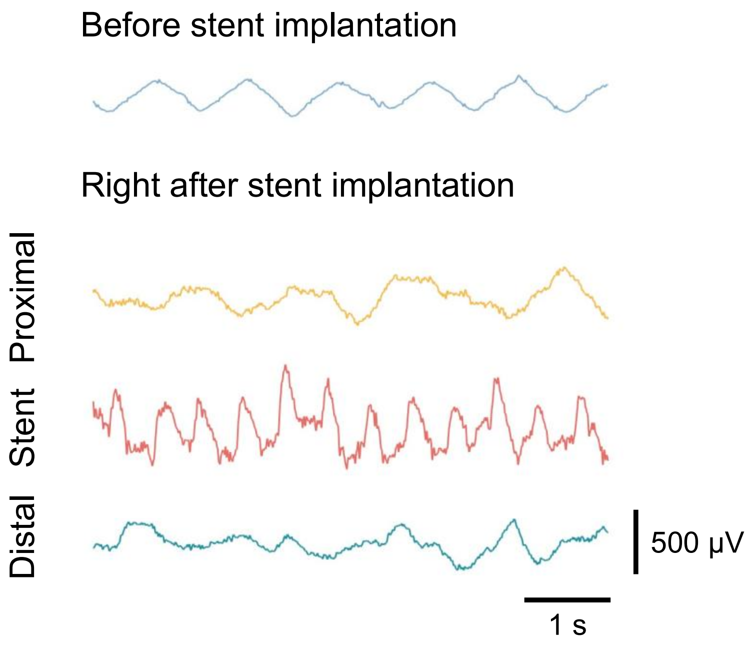

Supplementary Figure 24. Vascular electrophysiology recordings before and after stent implantation. Representative VE signals recorded before stent implantation (top) and right after stent implantation (bottom) from three positions: the proximal end, the stent site, and the distal end, respectively.

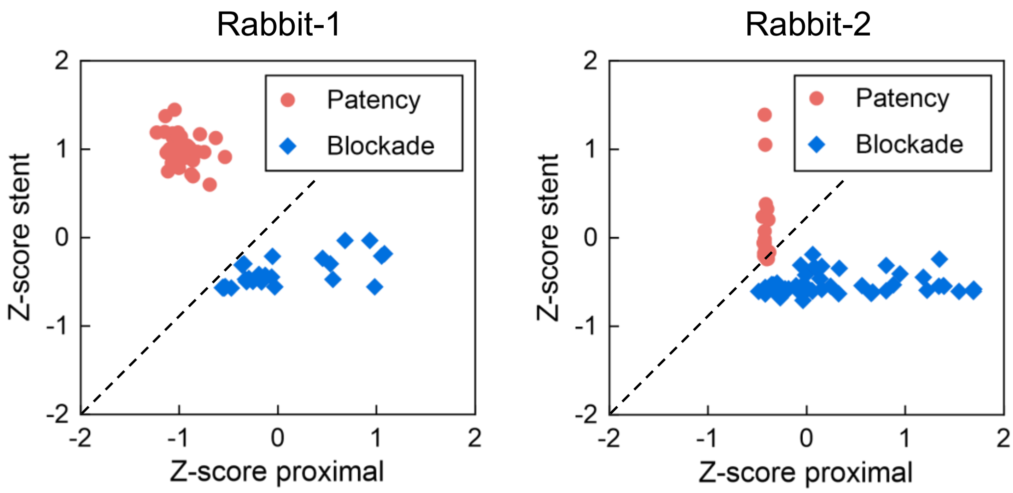

Supplementary Figure 25. Electrophysiological analysis of vascular stenosis. VE signals at the proximal segment and stent site were recorded immediately after stent implantation and one-month post-implantation. The power features of these signals were extracted and z-scored. A scatter plot of the z-score features at the stent site versus those at the proximal site illustrates the capability of electrophysiological analysis to distinguish between patent and stenotic vascular states.

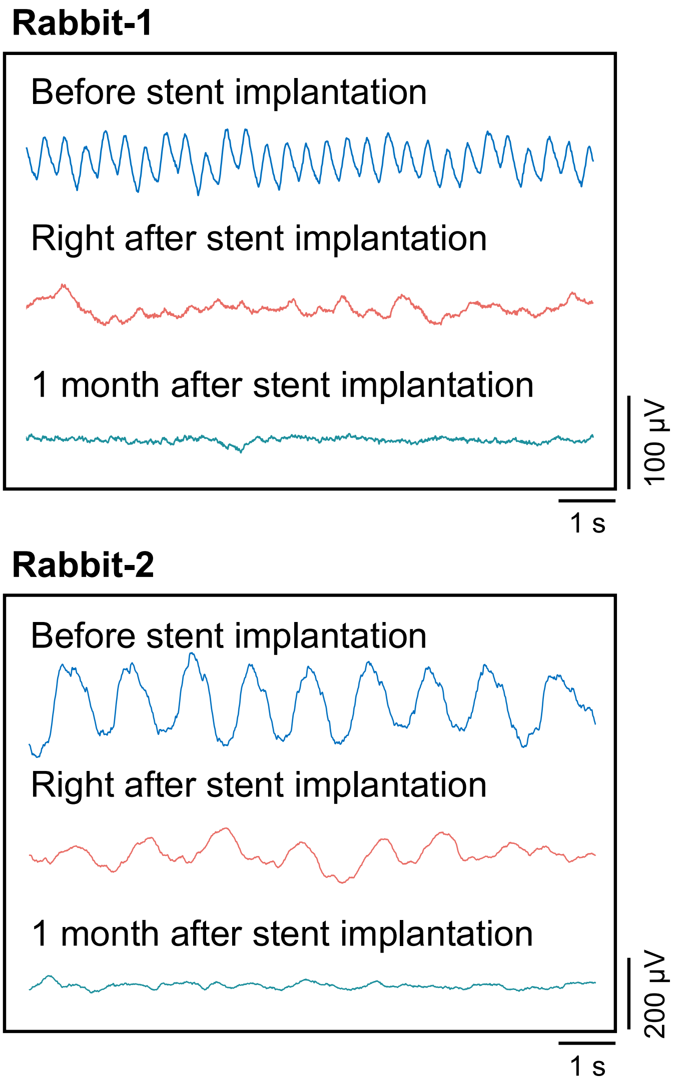

Supplementary Figure 26. Comparative analysis of VE signals with prolonged stent implantation duration. Representative time traces from one channel recorded through the BACE interface from the distal segment of the aorta at three critical time points: prior to stent implantation, immediately after stent implantation, and one-month post-implantation (left).

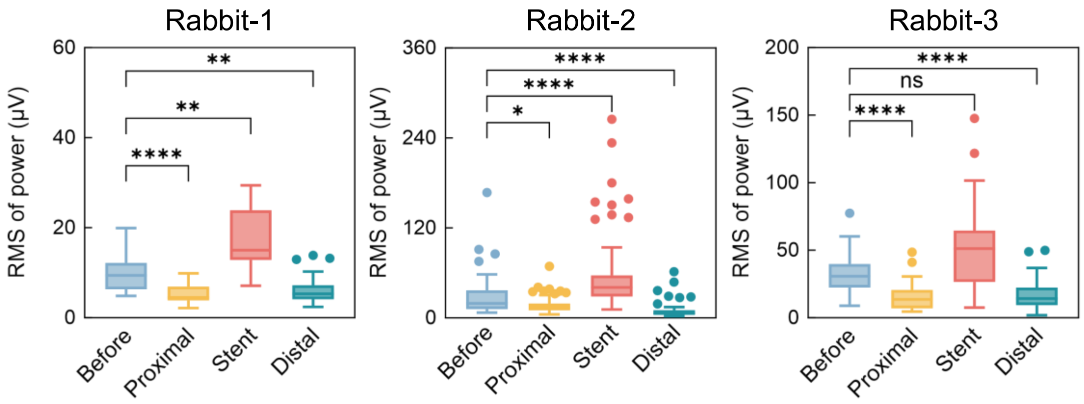

Supplementary Figure 27. Comparative analysis of the VE signals before and right after stent implantation from three rabbits. The VE signals were collected, as shown in Supplementary Figs. 24 and 26, and the RMS power features were extracted. Box plots represent the data median (center line), upper and lower quartiles (box bounds), 1.5 times the interquartile range (whiskers), and outlier values beyond this range (circles). p values for power comparison: $p = 4.29\times10^{-5}$ (Before vs. Proximal), $p = 2.08\times10^{-3}$ (Before vs. Stent), $p = 2.49\times10^{-3}$ (Before vs. Distal) for Rabbit-1; $p = 0.0448$ (Before vs. Proximal), $p = 8.50\times10^{-5}$ (Before vs. Stent), $p = 2.09\times10^{-10}$ (Before vs. Distal) for Rabbit-2; $p = 7.80\times10^{-8}$ (Before vs. Proximal), $p = 0.0694$ (Before vs. Stent), $p = 4.03\times10^{-6}$ (Before vs. Distal) for Rabbit-3. (ns: $p > 0.05$, *$p < 0.05$, **$p < 0.01$, ***$p < 0.001$, ****$p < 0.0001$, Kruskal-Wallis test with Dunn's post hoc test).

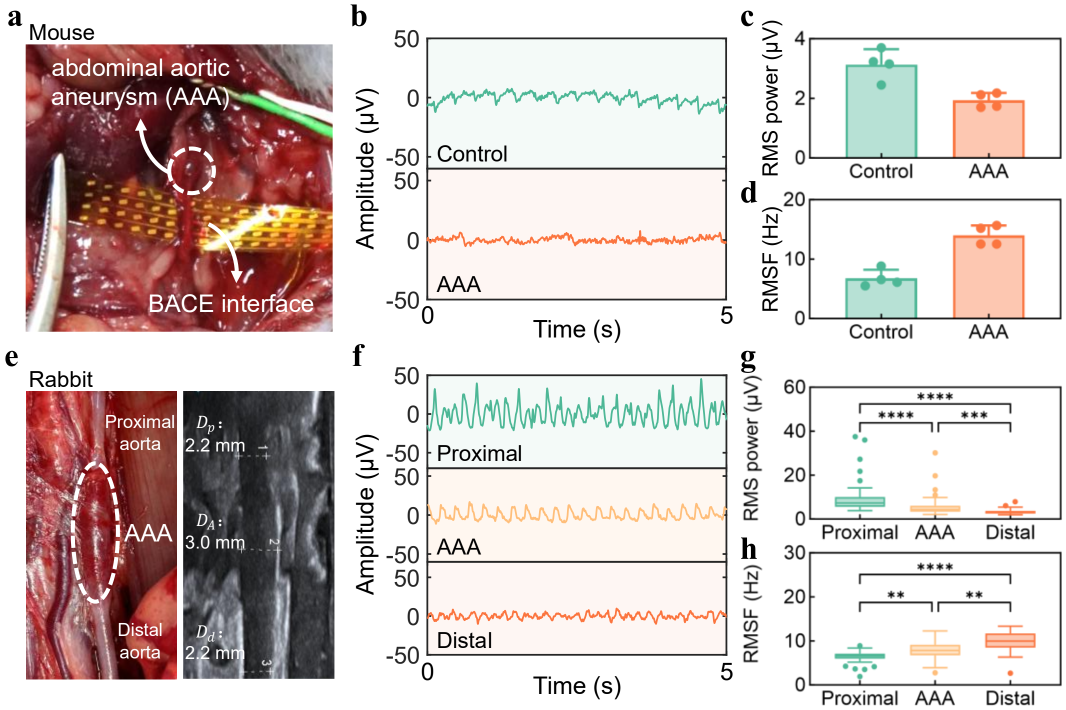

Supplementary Figure 28. Monitoring and comparison of vasomotor activity in AAA-affected aortas using the BACE interface. a, Photograph of the BACE interface implanted on the abdominal aorta of a mouse with abdominal aortic aneurysm (AAA). **b**, Representative time-domain traces of electrophysiological signals recorded from the control (healthy) and AAA aortas. Quantitative analysis of root mean square (RMS) power (**c**) and frequency (**d**) obtained from the electrical signals recorded from the control (healthy) and AAA aortas. **e**, Photograph of the exposed AAA in a rabbit model alongside its corresponding ultrasound image. Key anatomical landmarks including the proximal aorta, aneurysm region, and distal aorta are clearly annotated with measured vessel diameters. **f**, Representative time-domain vascular electrophysiological waveforms recorded from the proximal aorta, aneurysm site, and distal aorta using the BACE interface. Quantitative analysis of RMS power (**g**) and frequency (**h**) obtained from the electrical signals recorded from the proximal aorta, aneurysm site, and distal aorta. Data in **c** and **d** are presented as mean values ± SD. Box plots in **g** and **h** depict the data median (center line), upper and lower quartiles (box bounds), 1.5 times the interquartile range (whiskers) and outlier values beyond this range (circles). *p* values for RMS power comparison: $p = 4.798\times10^{-5}$ (Proximal vs. AAA), $p = 2.678\times10^{-15}$ (Proximal vs. Distal), $p = 5.831\times10^{-4}$ (AAA vs. Distal). *p* values

for RMS frequency comparison: $p = 0.0011$ (Proximal vs. AAA), $p = 3.223\times10^{-12}$ (Proximal vs. Distal), $p = 0.0011$ (AAA vs. Distal). (*$p < 0.05$, **$p < 0.01$, ***$p < 0.001$, ****$p < 0.0001$, Friedman test with Dunn's post hoc test).

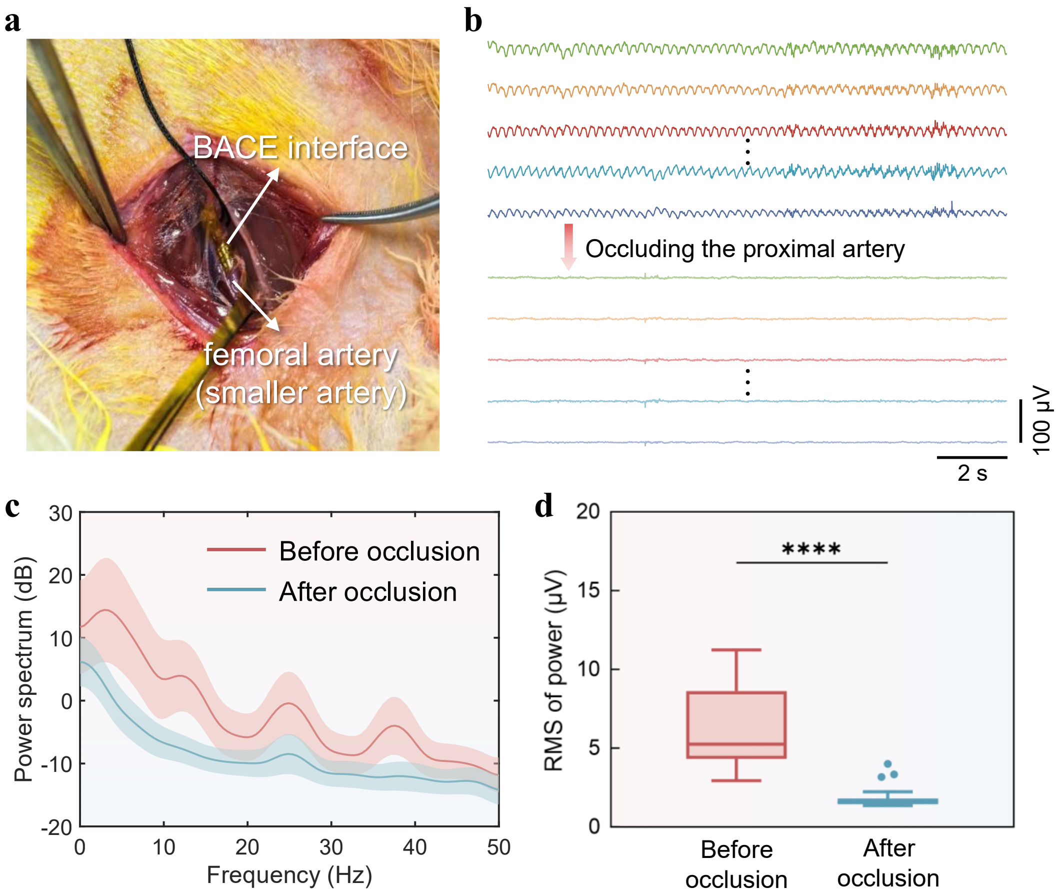

Supplementary Figure 29. Monitoring of vasomotor activity in the femoral artery under normal and occlusion conditions using the BACE interface. a, Photograph of the BACE interface implanted on the femoral artery of a rabbit. **b**, Time-domain traces of electrophysiological signals recorded from the femoral artery before and after inducing proximal artery occlusion (abdominal aorta ligation). **c**, Power spectral density (PSD) curves of the signals recorded from the femoral artery before and after proximal artery occlusion. **d**, Quantitative analysis of the RMS power showing significant changes in the signal following occlusion, reflecting altered vasomotor function. Data in **c** are presented as mean values ± SD. Box plots in **d** depict the data median (center line), upper and lower quartiles (box bounds), 1.5 times the interquartile range (whiskers) and outlier values beyond this range (circles). p values for power comparison before and after occlusion in **d**: $p = 1.1102\times10^{-16}$. (ns, : $p > 0.05$, *$p < 0.05$, **$p < 0.01$, ***$p < 0.001$, ****$p < 0.0001$, Wilcoxon signed-rank test).

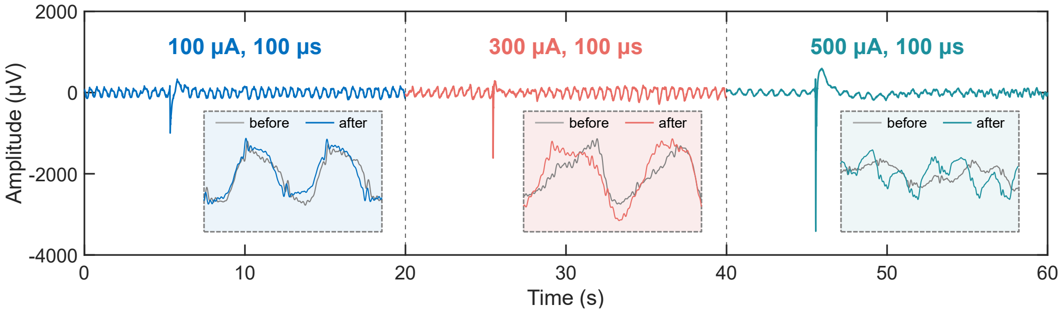

Supplementary Figure 30. Optimization and selection of stimulation pulse current amplitude in a rabbit without stent implantation. Charge-balanced biphasic current pulses of varying amplitudes with a pulse width of 100 μs were applied and compared. The results showed that 100 μA had no noticeable effect, while 500 μA induced phase distortion. In contrast, 300 μA yielded the most favorable results, which aligns relatively well with the findings in Fig. 4c.

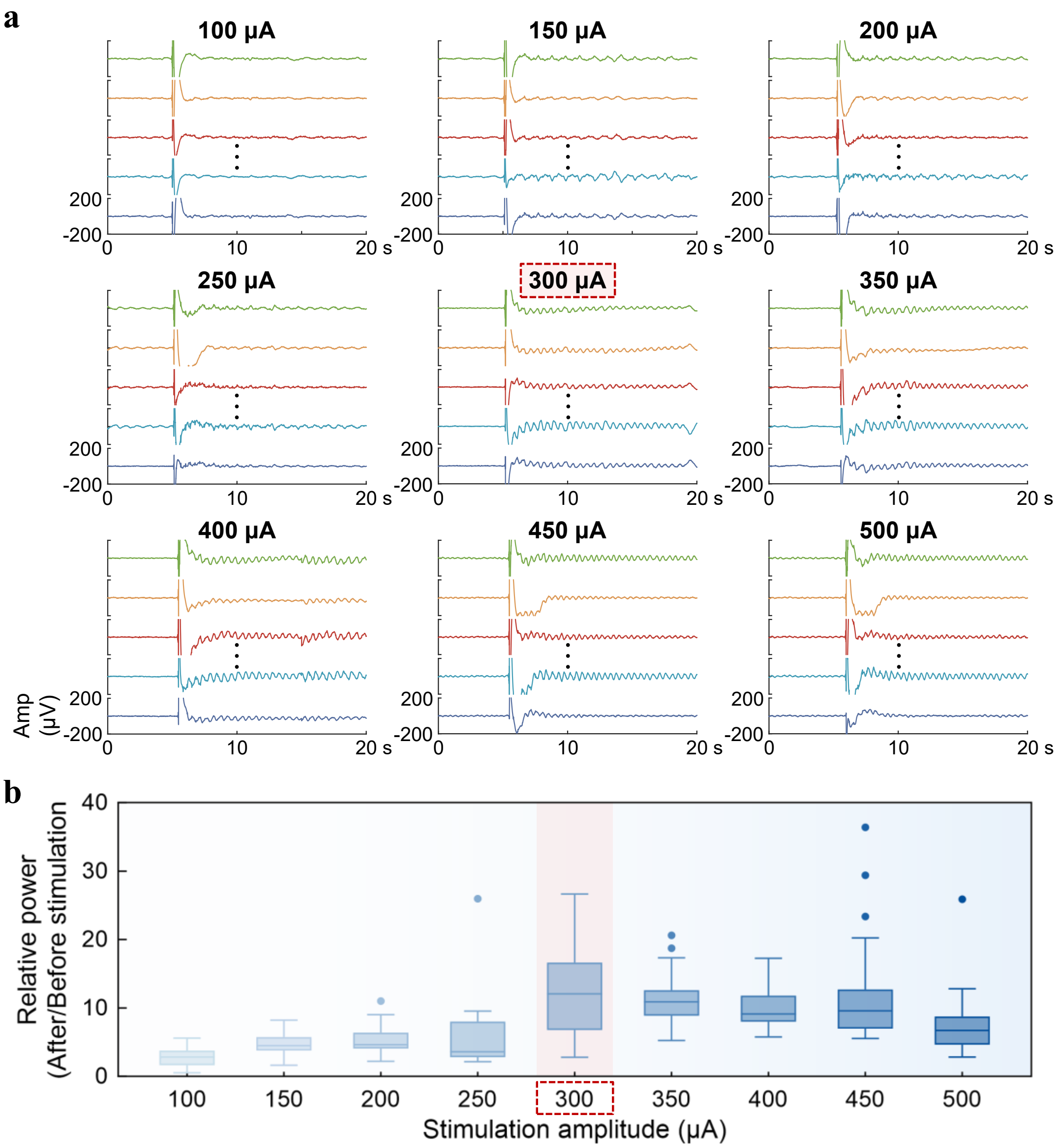

Supplementary Figure 31. Optimization of stimulation current amplitude for restoring distal vascular electrophysiological activity in stented arteries. a, Representative time-domain traces of electrical signals recorded from the distal aorta of stented rabbits following electrical stimulation at different current amplitudes ranging from 100 μA to 500 μA, in 50 μA increments. **b**, Quantitative analysis of relative signal power (post- vs. pre-stimulation) at each stimulation amplitude. The red shaded region and dotted box highlight the selected optimal amplitude of 300 μA. Box plots in **b** depict the data median (center line), upper and lower quartiles (box bounds), 1.5 times the interquartile range (whiskers) and outlier values beyond this range (circles).

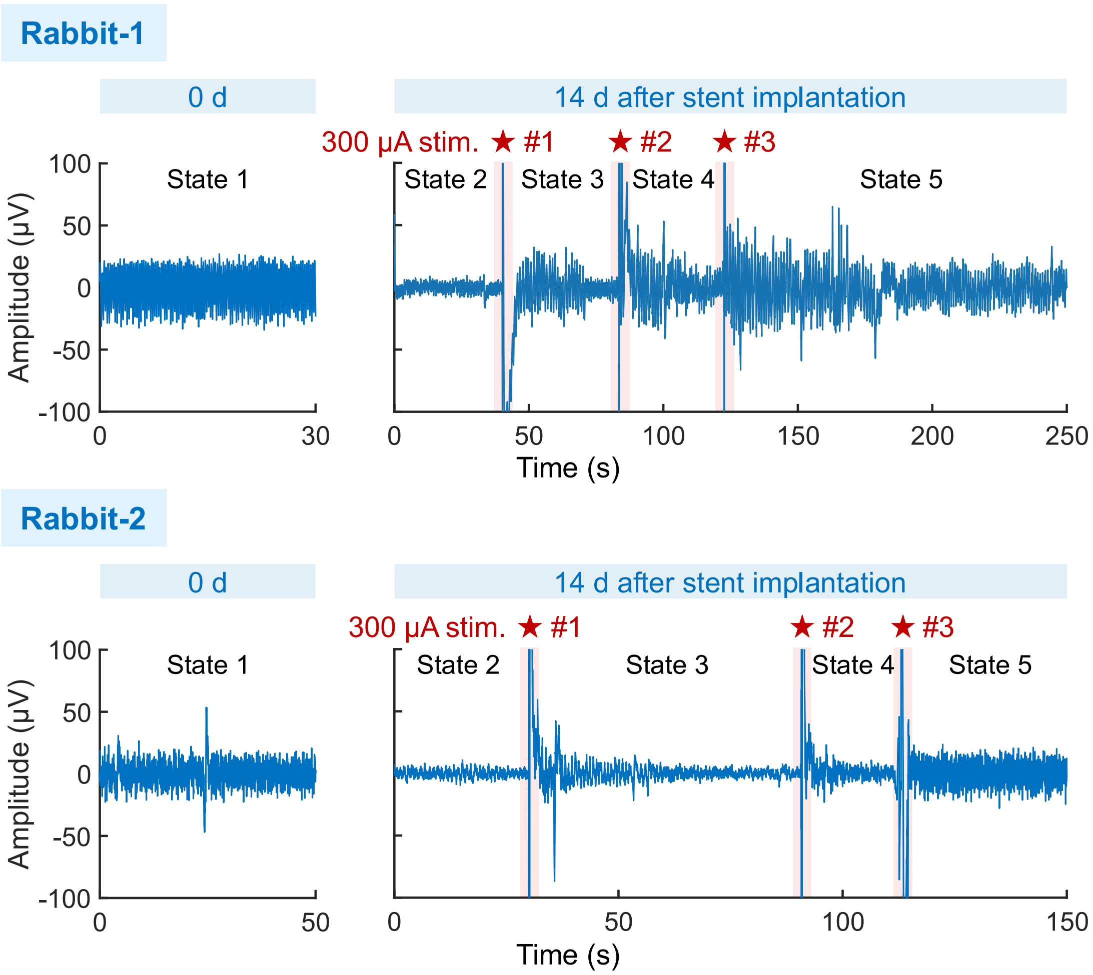

Supplementary Figure 32. Electrical regulation of vasomotor dysfunction. The VE signals recorded by the BACE interface from the distal segment of the artery in response to a series of electrical stimulations with a charge-balanced current pulse (pulse amplitude: 300 μA, pulse width: 100 μs) conducted 14 days after stent implantation from two rabbits. Red stars mark the timing of the stimulation events.

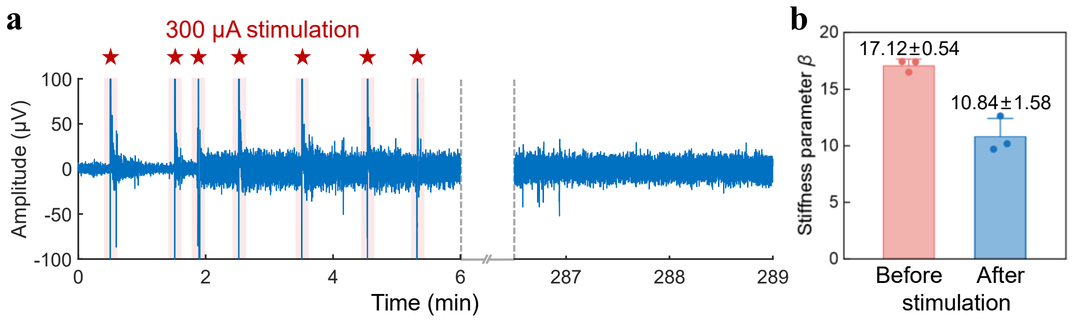

Supplementary Figure 33. Validation of the sustained effects of electrical stimulation. a, Electrophysiological response to intermittent 300 μA stimulation (indicated by red stars) over a prolonged period of time. **b**, The stiffness parameter β of the distal aorta before the first electrical stimulation and approximately 5 hours after the final stimulation. Data in **b** are presented as mean values ± SD.

Supplementary Table 1. Comparison of the sensing principles and performance with representative vascular mechanical sensors.

Sensor Type	Sensing Principle	Advantages	Limitations	Ref.
Piezoresistive	Resistance change due to mechanical strain	• Wireless monitoring of haemodynamics	• Susceptible to temperature drift<br>• Limited chronic stability<br>• Limited to physical measurements	[18]
Capacitive	Fringe-field-capacitive sensing	• Wireless monitoring of blood flow<br>• Biodegradable	• Susceptible to environmental electromagnetic interference and external noise<br>• Lack of direct electrical activity measurement	[19]
		• Wireless monitoring of diverse artery sizes and extents of occlusion		[20]
Piezoelectric	Piezoelectric effect to measure electromotive force caused by mechanical force	• Wireless monitoring of haemodynamics	• Susceptible to mechanical artefacts<br>• Lack of capabilities for electrical recording and modulation	[21]
		• Biocompatibility		[22]
Triboelectric	Contact electrification caused by mechanical motion	• Self-powered<br>• Bioresorbable	• Limited stability and repeatability<br>• Only mechanical sensing	[23]
**BACE interface**	Measure electrical signals generated by physiological processes	• Sensitivity to subtle electrophysiological signals<br>• Bidirectional, closed-loop recording and stimulation capability	• Lack of haemodynamics monitoring<br>• Wired configuration	**This work**

References

56. Kim, D.-H. *et al.* Dissolvable films of silk fibroin for ultrathin conformal bio-integrated electronics. *Nat. Mater.* **9,** 511–517 (2010).
57. Nawrocki, R. A. Super- and Ultrathin Organic Field-effect transistors: From flexibility to super- and ultraflexibility. *Adv. Funct. Mater.* **29,** 1906908 (2019).
