# Supplementary material for: Bioadhesive and conformable bioelectronic interfaces for vasomotoricity monitoring and regulation
Source: Nat Commun. 2025 Oct 14;16:9103. doi: 10.1038/s41467-025-64118-2 (PMC12521587; doi:10.1038/s41467-025-64118-2)
Supplement: Supplementary file 2 — Reporting Summary [file 41467_2025_64118_MOESM2_ESM.pdf]

nature portfolio

Corresponding author(s): Zhitao Zhou

Last updated by author(s): Jul 17, 2025

Reporting Summary

Nature Portfolio wishes to improve the reproducibility of the work that we publish. This form provides structure for consistency and transparency in reporting. For further information on Nature Portfolio policies, see our Editorial Policies and the Editorial Policy Checklist.

Statistics

For all statistical analyses, confirm that the following items are present in the figure legend, table legend, main text, or Methods section.

n/a	Confirmed	
☐	☒	The exact sample size (*n*) for each experimental group/condition, given as a discrete number and unit of measurement
☐	☒	A statement on whether measurements were taken from distinct samples or whether the same sample was measured repeatedly
☐	☒	The statistical test(s) used AND whether they are one- or two-sided<br>*Only common tests should be described solely by name; describe more complex techniques in the Methods section.*
☒	☐	A description of all covariates tested
☐	☒	A description of any assumptions or corrections, such as tests of normality and adjustment for multiple comparisons
☐	☒	A full description of the statistical parameters including central tendency (e.g. means) or other basic estimates (e.g. regression coefficient) AND variation (e.g. standard deviation) or associated estimates of uncertainty (e.g. confidence intervals)
☐	☒	For null hypothesis testing, the test statistic (e.g. *F*, *t*, *r*) with confidence intervals, effect sizes, degrees of freedom and *P* value noted<br>*Give P values as exact values whenever suitable.*
☒	☐	For Bayesian analysis, information on the choice of priors and Markov chain Monte Carlo settings
☒	☐	For hierarchical and complex designs, identification of the appropriate level for tests and full reporting of outcomes
☒	☐	Estimates of effect sizes (e.g. Cohen's *d*, Pearson's *r*), indicating how they were calculated

Our web collection on statistics for biologists contains articles on many of the points above.

Software and code

Policy information about availability of computer code

Data collection	Vascular electrophysiological data was collected by a multichannel data acquisition system CereCube NSP8 (Neuroxess Co., Ltd., China) and CereCube NSPS8 (Neuroxess Co., Ltd., China). CHI660E electrochemical workstation (CH Instruments Inc., China) was used for electrochemical data collection.
Data analysis	Vascular electrophysiology analysis was performed in MATLAB R2021a (MathWorks, Inc., USA). Statistical analyses were conducted using GraphPad Prism version 10.1.2 (GraphPad Software Inc., USA). The customized MATLAB scripts for electrophysiology analysis are available from Zenodo (https://doi.org/10.5281/zenodo.16017079).

For manuscripts utilizing custom algorithms or software that are central to the research but not yet described in published literature, software must be made available to editors and reviewers. We strongly encourage code deposition in a community repository (e.g. GitHub). See the Nature Portfolio guidelines for submitting code & software for further information.

Data

Policy information about availability of data

All manuscripts must include a data availability statement. This statement should provide the following information, where applicable:

- Accession codes, unique identifiers, or web links for publicly available datasets
- A description of any restrictions on data availability
- For clinical datasets or third party data, please ensure that the statement adheres to our policy

All data supporting the findings of this study are available within the article and its supplementary files. Any additional requests for information can be directed to, and will be fulfilled by, the corresponding authors. Source data are provided with this paper.

Research involving human participants, their data, or biological material

Policy information about studies with human participants or human data. See also policy information about sex, gender (identity/presentation), and sexual orientation and race, ethnicity and racism.

Reporting on sex and gender	No data related to sex and gender were collected and analyzed in this study.
Reporting on race, ethnicity, or other socially relevant groupings	This study did not involve any data related to race, ethnicity, or other socially relevant groupings.
Population characteristics	This study did not involve any human populations.
Recruitment	No recruitment of human participants was performed as this study did not involve human subjects.
Ethics oversight	As no human participants or biological materials were involved, no ethics oversight was required.

Note that full information on the approval of the study protocol must also be provided in the manuscript.

Field-specific reporting

Please select the one below that is the best fit for your research. If you are not sure, read the appropriate sections before making your selection.

☒ Life sciences ☐ Behavioural & social sciences ☐ Ecological, evolutionary & environmental sciences

For a reference copy of the document with all sections, see nature.com/documents/nr-reporting-summary-flat.pdf

Life sciences study design

All studies must disclose on these points even when the disclosure is negative.

Sample size	Sample size was determined based on previous reports on related topics to ensure sufficient sample sizes. For the in vitro characterization of the interface, the number of sample is 3-8, which is similar to the study (Deng, J., Yuk, H., Wu, J. et al. Electrical bioadhesive interface for bioelectronics. Nat. Mater. 20, 229–236 (2021). https://doi.org/10.1038/s41563-020-00814-2)). For the in vivo recording and modulation of the vascular electrophysiological activities, we used three biologically independent rabbits in each experiment, which is similar to the study (Liu, Z., Tang, C., Han, N. et al. Electronic vascular conduit for in situ identification of hemadostenosis and thrombosis in small animals and nonhuman primates. Nat Commun 16, 2671 (2025). https://doi.org/10.1038/s41467-025-58056-2; Yang, Q., Wei, T., Yin, R.T. et al. Photocurable bioresorbable adhesives as functional interfaces between flexible bioelectronic devices and soft biological tissues. Nat. Mater. 20, 1559–1570 (2021). https://doi.org/10.1038/s41563-021-01051-x).
Data exclusions	No data were excluded from the analysis in this study.
Replication	All attempts at replication were successful for the experiments. For the mechanical and electrical characterization of the interface, measurements were repeated on 3-8 individual samples. For the electrophysiological recording under varying degrees of vasomotor states, experiments were repeated with three independent biological replicates. In the stent implantation model, experiments at each time point were repeated across three independent biological replicates. The electrical stimulation experiments were repeated at least three times on each rabbit.
Randomization	In the stent implantation experiments, rabbits were allocated into three experimental groups based on the timing relative to stent implantation: pre-implantation, immediate post-implantation, and one month post-implantation. Within each group, animals were randomly assigned. For all other experiments, sample allocation into experimental groups was performed randomly.
Blinding	Blinding was not feasible due to the nature of the pharmacological or surgical intervention.

Reporting for specific materials, systems and methods

We require information from authors about some types of materials, experimental systems and methods used in many studies. Here, indicate whether each material, system or method listed is relevant to your study. If you are not sure if a list item applies to your research, read the appropriate section before selecting a response.

Materials & experimental systems

n/a	Involved in the study
☒	☐ Antibodies
☒	☐ Eukaryotic cell lines
☒	☐ Palaeontology and archaeology
☐	☒ Animals and other organisms
☒	☐ Clinical data
☒	☐ Dual use research of concern
☒	☐ Plants

Methods

n/a	Involved in the study
☒	☐ ChIP-seq
☒	☐ Flow cytometry
☒	☐ MRI-based neuroimaging

Animals and other research organisms

Policy information about studies involving animals; ARRIVE guidelines recommended for reporting animal research, and Sex and Gender in Research

Laboratory animals	The experimental New Zealand White rabbits, sourced from FMC Laboratory Animal Technology Co., LTD. (Zhejiang, China), were 18 weeks old, male, and weighed 3-3.5 kg.
Wild animals	No wild animals were used in this study.
Reporting on sex	To ensure consistency in the results, only male rabbits were included in the experiments.
Field-collected samples	No field-collected samples were used in this study.
Ethics oversight	The experimental protocol was approved by the institutional ethics committee of Huashan Hospital, Fudan University (Approval number: 202410031S).

Note that full information on the approval of the study protocol must also be provided in the manuscript.

Plants

Seed stocks	No plant-related experiments were involved, so the seed stocks were not applicable.
Novel plant genotypes	This study did not involve novel plant genotypes.
Authentication	This study did not involve plants requiring authentication.
